# The Experience of Goals and Rewards in Young People Who Self-Harm: A Qualitative Exploration

**DOI:** 10.3390/healthcare13243308

**Published:** 2025-12-17

**Authors:** Martina Di Simplicio, Ruksana Begum-Meades, Emily Gaardner-Bougard, Charis Eleftheriou, Oyinlola Akinsanya, Rachel Rodrigues, Lavanya Thana, Lindsay H. Dewa

**Affiliations:** 1 Division of Psychiatry, Department of Brain Sciences, Imperial College London, London W12 0NN, UK; 2 Division of Psychology and Language Sciences, University College London, London WC1E 6BT, UK; 3 NIHR Quality Safety and Outcomes Policy Research Unit, Yorkshire Quality and Safety Research Group, Bradford BD9 6RJ, UK; 4 School of Public Health, Imperial College London, London SW7 2AZ, UK

**Keywords:** self-harm, young people, goals, rewards, mental imagery

## Abstract

**Highlights:**

**What are the main findings?**

- Young people who self-harm describe an experience of anticipating rewards and setting goals largely characterised by over-valuing conditional enjoyment and distant unachievable goals.
- Young people who self-harm do not find imagining goal achievement a motivational tool, but rather an unhelpful escapism.

**What are the implication of the main findings?**

- More research is needed to understand how an imbalance between short-term and long-term goals may contribute to maintaining vulnerability to self-harm behaviour in young people.
- Helping young people to develop more adaptive rewards and goals, and to use mental imagery to support motivation, may represent a novel target for intervention in self-harm.

**Abstract:**

**Background/Objectives.** Self-harm is a heterogeneous behaviour with a lifetime prevalence of around 20% in young people aged 16–25 years old. Recent neurocognitive evidence suggests that, for some individuals, self-harm is associated with motivational processes similar to addiction, including maladaptive mental imagery, reward anticipation, and goal pursuit. However, our knowledge of young people’s subjective experiences of rewards and goals in relation to self-harm behaviour remains limited. Our study aimed to investigate how young people who self-harm experience enjoying and wanting rewards and pursuing goals in daily life and whether this experience changes during periods of self-harm. We also explored their use of mental imagery as a key cognitive process to support motivation. **Methods.** We conducted two parallel focus groups online (total of *N* = 12) with young people (mean age = 12.2, SD = 3; nine women, two men, and one non-binary) with a past-year history of self-harm behaviour. Qualitative data was analysed using inductive thematic analysis. Two young people with lived experience of self-harm informed the topic guide and data interpretation. Examples of questions were “Do you think your experiences of enjoying and/or looking forward to pleasant things are related in any way to self-harm, or not?” and “Do you visualise things you enjoy or may look forward to? If you do, how is that experience?”. **Results.** There were six themes: rewards need deserving, high self-standards, keeping control, trapped into long-term goals, unhelpful mental imagery, and self-harm alters the experience and anticipation of rewards and goal attainment. Most young people reported enjoying conditional rewards and working towards long-term goals that tend to be unattainable and beyond their control. Imagining these goals was experienced as unhelpful by most. For all young people, periods of self-harm thwarted enjoyment and goal achievement, shifted the preference to short-term immediate gratification, including from self-harm behaviour, and devalued long-term goals. However, our data cannot determine if these experiences are specific to young people who self-harm. **Conclusions.** Our findings indicate that the reciprocal relationship between motivational processes and self-harm behaviour in young people warrants further investigation. Helping individuals develop more adaptive rewards and goals, including appreciation of short-term goals and use of motivational mental imagery, could represent valued support for young people with self-harm.

## 1. Introduction

Self-harm is defined as an act of self-injury or self-poisoning, regardless of intent [1]. It is a complex and heterogeneous behaviour that typically peaks in frequency around age 16 years old [2]. Prevalence has increased over the past 20 years, with approximately 20% of young people under the age of 25 reporting having self-harmed at some point in their life [3,4]. Self-harm significantly increases the risk of suicide above the presence of other factors, including psychopathology [5,6]. Psychological interventions can help reduce self-harm, but access to therapy is limited, and their effect is often small [7,8].

Based on a recent meta-analysis, the most frequently reported functions of self-harm are to regulate emotions (likely prevalence 63–78%) and to escape aversive affect (62–78%) [9]. Consequently, existing interventions, such as cognitive behavioural therapy (CBT) and dialectical behaviour therapy (DBT), focus on improving emotion regulation and distress tolerance. However, it is well recognised that self-harm can also have other and multiple motives, including self-punishment (likely prevalence 41–62%) and communicating distress (30–55%) [9,10]. For some individuals, inducing a desired effect (42–57%) such as a sense of control, calm, or excitement can be an important driver of self-harm, leading to a positive reinforcement of the behaviour [11,12]. This highlights the need to personalise treatment to individual function(s) to improve outcomes [13,14].

The mechanisms maintaining self-harm may also be part of an addictive self-harm cycle [15,16], yet this is generally not considered in current treatments. Similarly to other addictive behaviours, the addictive experience of self-harm includes craving or urges to self-harm, anticipatory mental imagery related to urges, a loss of control over self-harm, increasing severity or frequency of self-harm over time, continuing despite negative consequences, and a cycle of abstinence and relapse [15,16].

The reward system is key in maintaining addictive behaviours [17,18] and is an area of interest in the maintenance of self-harm. Some studies have shown that at a neurocognitive level, differences in the processing of rewards (namely, anticipation synonymous with ‘wanting’ or craving, and consummation synonymous with ‘liking’ or enjoying rewards) are associated with self-harm thoughts and behaviour [19,20,21]. However, the theoretical implications of this are unclear. One hypothesis posits that a heightened neural consummatory response to rewards may contribute to engagement in risky behaviours such as self-harm, particularly in adolescence [19]. Another hypothesis, drawing on the addiction literature [17,18], posits that a reduced neural response when anticipating common rewards such as money [20] may lead to seeking out other more salient rewards such as self-harm. This putative anticipatory neural bias may be secondary to concurrent depressive symptoms such as anhedonia but nevertheless represent a process that may directly contribute to maintaining self-harm in a similar way to an addiction [21].

Mental imagery is the experience of “seeing or hearing through the mind’s eye” in the absence of an external sensory stimulus [22]. Imagining what one desires is a key component of motivational processes, i.e., of anticipating, wanting, and working towards rewards [22,23]. Mentally simulating enjoyable scenarios boosts anticipated pleasure [24] and impacts on actual behaviour [25,26]. On the other hand, poor reward-related mental imagery contributes to reduced effort towards achieving goals [27] and to depressive symptoms [28,29]. There is growing evidence that mental imagery of self-harm contributes to wanting to self-harm [30], similar to mental imagery of substances in addiction [31]. However, the motivational mental imagery of general rewards, of desired outcomes, and how to achieve these remains unexplored in relation to self-harm. Finally, difficulties in self-regulating goals, another process closely related to reward and motivation, are also present in individuals who self-harm [32,33]. Difficulties in vividly imagining enjoyable activities or the accomplishment of goals could hinder efforts at engaging in alternative behaviour to self-harm. Interestingly, positive mental imagery combined with goal-setting interventions in young people who self-harm can lead to improved emotional wellbeing and sense of control [34]. We have recently shown that motivational imagery of adaptive behaviours can reduce the frequency of self-harm [35,36] and improve goal achievement [37].

In summary, growing evidence suggests that dysfunctional motivational processes may be involved in self-harm behaviour and represent so far neglected therapeutic targets. While previous research has examined similarities between addiction and self-harm behaviour at the subjective level [38,39,40], the subjective experience of motivational processing (liking, wanting) in self-harm has not yet been investigated. Understanding how young people who self-harm experience everyday reward-processing and goal setting is key to (a) guide further mechanistic research and (b) ground intervention development in what is relevant to young people. This study aimed to explore how young people who self-harm anticipate and experience rewards, set goals, and experience goal pursuit. We also aimed to explore how young people use mental imagery to simulate rewards and goals, as mental imagery is a key cognitive process supporting motivation. Our research question was the following: how do young people who self-harm experience rewards and goals in their daily life?

## 2. Materials and Methods

### 2.1. Design

We adopted an interpretivist approach to gain an in-depth understanding of young people’s views on reward and goal-pursuit behaviour in relation to their lived experiences of self-harm. However, we recognise that while we consciously endeavoured to keep an open stance, our approach was influenced by the quantitative neurocognitive research conducted by our team. Given the absence of previous qualitative studies on this specific topic and the heterogeneity of self-harm presentations, the choice of focus group format meant we could interact with participants and be exposed to different viewpoints, facilitating a broader exploration of themes and depth of discussion. The consolidated criteria for reporting qualitative research (COREQ) checklist guided our reporting.

### 2.2. Participants and Procedure

A purposive sample of participants from the experimental study IMAGINE investigating motivational biases in young people with self-harm were recruited (see details in Yavuz, Rodrigues et al., 2024 [41]). The IMAGINE study recruited young people from the general population aged 16–25, fluent in English language, and with at least one episode of self-harm within the past year. Exclusion criteria were current psychotic symptoms, acute severe suicidal ideation, or any severe neurological impairments. Young people who took part in the IMAGINE experimental session and consented to take part in qualitative focus groups were contacted via email and provided with a participant information sheet. None of the recruited participants were personally acquainted with the researchers who were present during the focus groups. Recruitment continued until an adequate number and diversity of representation (e.g., age, gender, and ethnicity) was achieved within the study time limits. A convenient day and time for the focus groups was then agreed among those who had expressed an interest. Written consent was taken electronically prior to the focus groups arranged date. Each participant was reimbursed GBP 40 for their time.

### 2.3. Topic Guide

Topic guide development was informed by a pre-registered systematic review (PROSPERO registration CRD42019132053) investigating the presence of reward processing in self-harm behaviour measured at cognitive, behavioural, and/or neurobiological level. First, a new search was conducted to look for relevant qualitative studies on reward processing in self-harm from three electronic databases (EMBASE, PsycINFO, and MEDLINE) on 21 March 2021 using the pre-registered criteria. Two independent raters (CE, OA) screened the result of the articles (inter-rater reliability = 82%) and extracted 19 qualitative papers for full-text screening. No paper addressed the pre-registered research question, “Do reward processing biases exist in self-harm?”, and so no data extraction was conducted. Related concepts from the full-text papers and categories from the pre-registered review were then used to inform the nature of the questions and prompts in the topic guide. To minimise the risk of introducing bias derived from interest in reward processing and addiction models of self-harm, we deliberately refrained from directly asking participants about causal relations between motivational processes and self-harm. The topic guide was then reviewed, critiqued, and finalised by two members of a Young People Advisory Group (YPAG—see below), before being finalised for use in the focus groups (Table 1).

### 2.4. Data Generation

The number of participants was guided by thematic saturation [42], whilst also constrained by academic timelines. Thematic saturation was broadly reached, with recurring patterns around reward anticipation, goal-pursuit, and control consistently observed across participants in the two groups, ensuring sufficient depth and richness of data for the study’s focused aims. Two parallel focus group discussions were conducted via Zoom in April 2021 to explore participants’ subjective experiences of rewards, goal pursuit, and mental imagery in relation to self-harm. Each group participated in two 90 min sessions (S1 and S2) to cover the entire topic guide, allowing enough time for discussion. This method allowed participants to share and reflect on their experiences, consistent with the interpretivist aim of understanding meaning in context. Participants were allocated to Group 1 or 2 based on their time and date availability. We aimed for 6–8 participants per group [42], a sample size that would balance individual contribution with capturing a range of perspectives. Two participants invited to Group 2 did not attend S1, resulting in uneven numbers between the groups. Sessions were co-facilitated by CE, OA, and MDS, beginning with introductions and ground rules. Participants discussed their experiences, while facilitators prompted for depth and clarity. Non-verbal behaviours, tone, and emotions were noted for contextual insight (e.g., to assess relative agreement between participants around each sub-theme). All sessions were audio-recorded, transcribed verbatim, anonymised using participant IDs, and destroyed after transcription. Data handling followed GDPR guidelines to ensure confidentiality and ethical compliance.

### 2.5. Data Analysis

Data were analysed using reflexive thematic analysis following Braun & Clarke’s updated guidance [43], emphasising reflexivity, rigorous coding, and transparency in the analytic process. Deductive elements informed by neurocognitive models of reward and motivation also influenced our final data interpretation. Transcripts were first read and re-read by the research team to ensure familiarisation with the content. Preliminary codes were then generated inductively from the raw data, capturing meaningful features of the discussions. Coding was carried out independently by two researchers (CE, OA), with discrepancies discussed until consensus was reached. Codes were collated into sub-themes, which were then conceptualised into tentative themes. These candidate themes were reviewed in collaboration with the two members of the YPAG and refined through iterative discussions with the full research team, enabling analyst triangulation and enhancing interpretive depth. Transcripts were not re-sent to participants for member checking.

Reflexivity was integral to the analytic process, particularly given that one member of the research team and two members of the YPAG were themselves young people with lived experience of self-harm. This positionality brought valuable insider insight into the language, context, and emotional tone of the discussions, supporting a more nuanced understanding of participants’ accounts. At the same time, we discussed how shared experiences and similarities in age could introduce assumptions or unnoticed points of resonance that might shape interpretation of the data. In parallel, research team members contributed a clinical, psychiatric, and cognitive neuroscience perspective, offering insight into mental health, self-harm behaviours, and broader psychological/neurobiological frameworks. The combination of insider (lived experience) and professional perspectives allowed for a balanced interpretation, highlighting both contextual richness and theoretical relevance.

### 2.6. Patient and Public Involvement

Two young people with lived experience of self-harm informed the topic guide design and analysis research stages (two females, one BAME, one neurodivergent) via two meetings lasting approximately 60 min, one prior to data collection (March 2021) and one after data coding (April 2021) on Microsoft Teams with members of the study team (MDS, CE and OA). The young people were members of the YPAG supporting the IMAGINE study (see Rodrigues et al., 2023 [44]), which had seven members in total recruited via social media and local networks and advised the study between 2018 and 2023 via online and in-person meetings.

## 3. Results

### 3.1. Sample

Twelve young people participated across two focus groups. Demographic characteristics are reported in Table 2. Mental health characteristics (depression scores and self-harm frequency) were collected during the IMAGINE study experimental session, which took place approximately 6–12 weeks prior to the focus groups, and so may not accurately reflect the experiences of the participants at the time of the focus groups.

### 3.2. Themes

**Figure 1:**
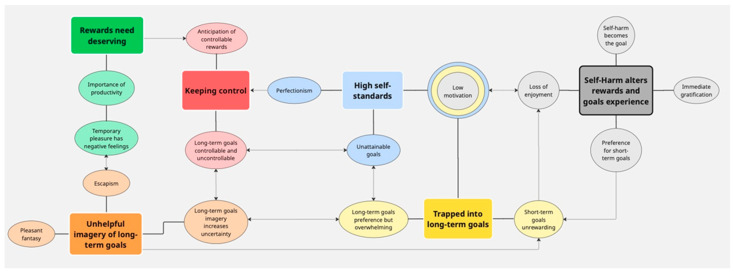
Thematic map illustrates how different themes and sub-themes are connected to form young people’s experience of rewards and goals, and how this is perceived to change in relation to self-harm. Themes are in rectangular boxes, with respective sub-themes in oval boxes of the same colour. Low motivation sub-theme has multiple colours to indicate that it is repeated across three themes. Continuous lines connect each theme with its sub-themes. Thin lines depict links between different themes and their sub-themes, with arrows suggesting directionality of the link. E.g., Undervaluing short-term goals may contribute to loss of enjoyment when short-term goals are preferred; if imagery increases sense of uncertainty around long-term goals, it may also increase the perception that long-term goals are overwhelming. Conversely, imagery may be unhelpful because imagined long-term goals are uncontrollable and overwhelming.

Six major themes were identified: rewards need deserving, keeping control, high self-standards, trapped into long-term goals, mental imagery of long-term goals not helpful, and self-harm alters the experience and anticipation of rewards and goals attainment. The themes are summarised together with sub-themes in Table 3 and conceptualised in a thematic map (Figure 1). Themes are illustrated with quotes below (P = Participant; G = Group; S = Session).

#### 3.2.1. Theme 1: Rewards Need Deserving

All participants were able to describe things that they enjoy and consider rewards, such as activities and hobbies. In particular, gaining a reward because of their own productivity and hard work provided all participants with feelings of satisfaction and gratification. In contrast, most participants expressed guilt for enjoying a reward (like a compliment or money) if they felt they did not deserve it or had not earned it.

*“If I just do something I enjoy without doing something productive, you could say, it would be at the back of my head that I’m doing something, but I shouldn’t be doing it…”*

(P5, G1, S1)

A few participants went all the way to describe activities which might have been experienced as temporarily enjoyable (such as watching videos on YouTube or TV series) as unproductive “avoidance behaviours”, ultimately associated with negative emotions. However, a few others disagreed with the need to deserve rewards to enjoy them.

*“When it [the enjoyable activity] is a bit more avoidance it feels good at the time but not really like after”*

(P9, G2, S1)

*“Nah (laughs) I just feel like it’s a tough life and you have to have nice things and do things that make you happy. You deserve it regardless.”*

(P1, G1, S1)

#### 3.2.2. Theme 2: Keeping Control

For most participants, perceived control over future rewards was very important. For some, when they felt a lack of control over a future reward or goal, this had the potential to generate negative reactions, such as feeling anxious. For others, having no control over delayed gratification led to feeling stressed. Consequently, these participants expressed only anticipating future rewards if they felt that they had control over obtaining them.

*“I just really think it [looking forward to something] depends on how much in control of this thing I am and how much certainty there is because I find it really hard with like kind of unpredictable, uncertain things”*

(P9, G2, S1)

*“If I really am looking forward to it then I want it sooner like really badly—I’m not prepared to wait kind of thing so it can be quite uncomfortable and quite stressful if it is quite far away.”*

(P3, G1, S1)

The need for control translated into varying attitudes towards short-term (usually more controllable) vs. long-term anticipated rewards. Short-term rewards felt more controllable to some participants: these would be more mundane events (like seeing friends or receiving a parcel), leaving space for excitement and positive anticipation. In contrast, long-term rewards and goals were described more in the domains of “life goals”, and their uncertainty was perceived by most as less controllable and anxiety-provoking. However, a few participants viewed this uncertainty also as offering a chance of exerting some control and shaping the future.

*“I look at like what I have next week to look forward to because I’m really scared of the future. So next week, even if it’s like going out with friends, I’d look forward to that, but I’m not really like far-fetched.”*

(P3, G1, S1)

*“I look forward to things much further into the future maybe because it’s uncertain so I can just shape it however I want”*

(P7, G1, S1)

#### 3.2.3. Theme 3: High Self-Standards

Most participants reported a tendency for high self-standards, which translated into goals that were described as too many, unrealistic, all-or-nothing, and overwhelming. A few termed the high standards perfectionism and saw it as leading to positive experiences and achievements. Most participants, however, mentioned the potential negative impact of their high standards on their own motivation and the ability to put effort into achieving a goal. Participants expressed a consensus that high standards could deplete energies and have a downstream negative effect on general mental health.

*“My experience of goals is difficult because I set myself extremely unrealistic goals and then destroy myself trying to get to them”*

(P10, G2, S2)

*“I set all these goals and didn’t do anything so I sort of blame myself because I’m not doing these goals, I’m not progressing so it’s kind of a negative thing in a way, too many goals”*

(P5, G1, S2)

#### 3.2.4. Theme 4: Trapped into Long-Term Goals

All participants were able to identify goals and how both setting and pursuing them can be affected by internal factors including mood, fear of failure, and external factors such as sharing a collective goal (e.g., preparing an exam) or receiving other people’s validation (e.g., parents’ feedback or a good mark). Most participants tended to dismiss short-term goals and their ability to produce any sense of reward. This was explained as the small steps leading to a bigger goal not counting as sources of gratification or anticipation.

*“I just don’t view anything short-term as valuable, it’s like always working towards the next big thing”*

(P10, G2, S2)

*“I reckon the term goals is something a bit bigger so just like P5 and P2 said with having a to do list. I’m not gonna call them ‘small goals’.”*

(P1, G1, S2)

All participants described that long-term goals such as career or relationship aspirations were the important and rewarding ones. However, all also noted that the pursuit of long-term goals could generate a sense of being exhausted and overwhelmed because these goals are big, important, and cannot be achieved quickly. For some participants, this meant they lost motivation towards long-term goals due to their unattainability or to the absence of immediate gratification. Only a few participants described turning to pursuing short-term goals.

*“A lot of the time when I’m setting big goals, I become unmotivated because it seems so overwhelming like for example, I wanted to be a clinical psychologist since like sixth form but because it was so overwhelming and such a big thing, I became so overwhelmed and just didn’t want in the end, so I lost sight of that goal”*

(P2, G1, S2)

*“Because a long-term goal just seems so unachievable, I just sort of ignore them and they no longer feel like goals [be]cause there is like no clear plan to work towards them so I kind of prefer short term goals”*

(P7, G1, S2)

#### 3.2.5. Theme 5: Mental Imagery of Long-Term Goals Not Helpful

Two participants reported never visualising things in their mind. All other participants described imagining long-term (rather than short-term) rewarding scenarios and goals. For them, imagining long-term goals was potentially distressing because it amplified the sense of uncertainty and unattainability, fear of failure, and feelings of sadness or frustration for “not getting there”. Most participants viewed imagining achieving goals as a form of escapism not conducive to goal attainment, and some participants explicitly named positive mental imagery as wasting time instead of finding a solution to one’s problems.

*“When I imagine positive things, it’s like things very distant in the future, like whether it’s my dream career or having a house”*

(P7, G1, S2)

*“I agree it makes me feel sad and overwhelmed and it’s almost like this is how life could be and you’ve made some rubbish choices so you’re never gonna get there so it actually makes me feel a bit rubbish”*

(P11, G1, S2)

While agreeing with the above, a few participants also reported that at times, picturing future goals in their mind was pleasant or comforting. These participants described that simulating desired scenarios would be helpful to lift mood temporarily, to feel prepared, or to increase motivation. However, they also experienced that the positive effects of imagery did not last or elicited ambivalent feelings.

*“So when I do my whole like, rehearsing-imagining that feels like it’s actually really productive because I feel like I’ve prepared myself for something so that I definitely feel a sense of reward.”*

(P9, G2, S2)

*“It feels comforting and enlightening but also sad for me because I’m like I don’t know if I’ll ever get there”*

(P1, G1, S2)

#### 3.2.6. Theme 6: Self-Harm Alters the Experience and Anticipation of Rewards and Goal Attainment

During periods of self-harm, some participants described losing the ability to enjoy or to look forward to things they usually find pleasant. Some participants reported that activities that are usually enjoyable and typically used as positive coping mechanisms were no longer pleasant, as they required effort that would not pay off and/or did not provide immediate gratification. The latter experience translated for some into seeking more gratifying rewards from more energetic, harder to achieve, or more intense activities.

*“I feel like nothing could cheer me up [during times of self-harm] I guess you could say”*

(P5, G1, S1)

*“When I’m really not feeling great I struggle to be patient and I just feel like I need to feel different right now and that’s why I find it much harder to get myself to engage in these positive things cause I know that it will be effort and I won’t necessarily get something back for my effort straight away.”*

(P12, G2, S1)

All participants agreed that in periods of more frequent self-harm, goals would shift from long-term to short-term. For some participants, the focus on short-term goals was seen as a means of survival; for others, it reflected instead the search for immediate gratification. Most participants explicitly mentioned low mood as an important concurrent factor with self-harm that influenced seeking immediate rewards. Participants referenced the previous discussion around long-term goals and expressed how low motivation made them refocus on short-term goals. In this context, a few reported the use of positive imagery of the future to alleviate negative affect, framed as an escape from the difficult reality.

*“Erm, yeah, I think I would choose things that provide a lot of gratification but don’t require much effort. Other times, simple tasks that make me feel like I’ve achieved something like a short workout or doing groceries—something you can tick off a list or you know, I don’t tend to focus on long-term goals. They just seem unachievable, and I’m not motivated towards achieving them”*

(P5, G1, S2)

*“Yeah, I would discard the long-term goals and focusing on the short-term ones, so you feel like the effects quicker—so more like a to-do list like we said earlier”*

(P3, G1, S2)

Some participants also reflected that self-harm could become the epicentre of goal pursuit, either to obtain the reward from self-harm or to stop self-harming.

*“Rather than looking forward to things in the future, it would always be looking forward to my next incident of self-harm.”*

(P2, G1, S1)

*“I would only have one goal—don’t cut”*

(P8, G2, S2)

## 4. Discussion

Our study examined for the first time the subjective experience of rewarding activities and goal pursuit in young people who self-harm, with a particular interest in the use of mental imagery. Young people expressed six main themes: rewards need deserving, keeping control, high self-standards, trapped into long-term goals, mental imagery of long-term goals not helpful, and self-harm alters the experience and anticipation of rewards and goals pursuit. We found that overall young people with self-harm looked forward to and enjoyed rewards in their daily lives. However, anticipation and enjoyment tended to be conditional on productivity and a sense of control over obtaining rewards. Young people with self-harm often worked towards goals that they perceived as unattainable, while paradoxically also reporting a need for control over these goals. Long-term goals were preferred over short-term ones; however, this preference was reported to shift during periods of self-harm, with the desire for immediate gratification becoming clearer. The use of mental imagery appeared to be limited to anticipating long-term goals and was largely reported as being unhelpful and, at times, distressing.

Young people in our study did not report any difficulty in anticipating or embracing (wanting and liking) rewarding experiences in their day-to-day lives. However, during periods of self-harm, they reported a lack of motivation and inability to enjoy or look forward to pleasant experiences, which they associated with depressed mood, consistent with previous literature on future-thinking, reward, and motivational biases in depression [45,46,47,48]. These findings are in line with experimental and neurobiological evidence showing individuals who self-harm have a typical or enhanced response to consummation of rewards [19,49,50,51,52], and that reduced anticipation of rewards in those with self-harm is associated with depressive symptoms [20]. Critically, however, any association between this reward bias and craving for self-harm at the subjective level was unclear from our findings and should be explored in future studies.

Another characteristic endorsed by most participants was the dismissal of temporary, short-term rewards, which could instead generate distress unless perceived as “productive”. The need for deserving rewards may reflect the ubiquitous emphasis on productivity in Western culture beyond any association with poor mental health or self-harm. Low self-esteem, poor self-compassion, and self-punishment are often endorsed by young people who self-harm [53] and may also drive this refusal of purely hedonistic or fortuitous gratifying experiences. In contrast, during periods of self-harm, participants’ focus shifted to short-term goals as a means of survival to get through the day or avoid self-harming, while working towards long-term goals became less relevant. However, some previously rewarding activities (e.g., going for a walk) became less appealing if they required effort or if they lacked intensity, suggesting that the shift to small achievable goals during periods of poor mental health does not always focus on adaptive behaviour in young people with self-harm. In fact, for some young people, self-harming would become the only achievable and overwhelming goal in their day.

Another theme expressed by participants was the importance of perceived control over rewards and goals. Having an external locus of control, whereby an individual believes their actions and environment are determined by external forces, has been described as a personality trait characteristic of individuals who self-harm in correlational studies [54,55]. It may be that young people in our study expressed the need to be in control to counteract an external locus of control. Similarly, the need to regain control is often described as a driver of self-harm as part of emotion regulation [56,57]. However, previous neurobiological research has also suggested that control enhances the activation of reward processing regions in the brain [58,59]. Future research could elucidate whether the need for control is a generalised trait or an automatic mechanism to heighten blunted reward anticipation in this population [20].

The theme of high standards and perfectionism has previously been associated with self-harming behaviour [60]. In our findings, this trait appears to drive the focus towards ambitious goals that are nonetheless perceived as unattainable, consistent with previous research in cohorts of self-harming adolescents [61,62]. It is possible that goals are perceived as unattainable due to self-criticism [62], pessimistic biases [63,64] and low self-efficacy [65], all strongly associated with poor mental health [66] and not necessarily specific to self-harm. However, the combination of high standards, low attainability, and overvaluation of long-term goals may lead to feeling trapped, one of the key motivational factors in the Integrated Motivational Volitional model of self-harm [67]. For example, some participants described ‘painful engagement’, i.e., the inability to relinquish unattainable goals, which can compromise motivation for new goals [64]. Young people in our study expressed the pitfalls of pursuing long-term goals, including overwhelm and exhaustion [63,66]. However, they appeared to dismiss short-term goals outside of periods of self-harm and also denied imagining short-term goals. It would be important to understand if and how lack of mental imagery and devaluation of short-term goals are related, and if they are typical of those who self-harm, given evidence from the general population that adolescents may be more sensitive to immediate, short-term rewards compared to long-term goal setting [68].

Critically, for young people with self-harm, the use of mental imagery was limited to distant goals and was overall found unhelpful and distressing as it amplified the sense of uncertainty and unattainability. A previous qualitative study described that adolescents with anhedonia have difficulties imagining pleasurable events in the near future but still look forward to distant goals [69]. Among our participants, even when imagery conveyed anticipated pleasure and satisfaction, it appeared to subsequently generate the cognitive dissonance between present and desired self [70] or was viewed by most as merely escapism. However, it is possible that undetected aphantasia within the sample and/or effects of age on the ability to generate complex mental imagery (which may be underdeveloped in adolescents) skewed our findings [71]. As mental simulation of proximal activities contributes to adaptive planning and motivation [72,73,74], future studies should test whether a lack of effective future goal-attainment imagery directly impacts reduced goal specificity, goal expectancy, low motivation, and effort towards goal achievement reported in individuals with self-harm [63,75]. When reducing self-harm is a goal, the development of adaptive goal-directed motivational imagery could then represent an important target for intervention in this population, as suggested by initial findings from our Imaginator studies [35,36,37]. Crucially, Imaginator focuses on proximal rewarding goals that offset an individual’s specific function of self-harm.

### 4.1. Strengths and Limitations

A key strength was that we built qualitative research questions on a systematic review framework (https://www.crd.york.ac.uk/PROSPERO/view/CRD42019132053, accessed on 14 December 2025) and with lived experience input, directly seeking to add rich qualitative insight to previously conducted quantitative studies in this area. The involvement of multiple analysts and iterative review of themes ensured that insider perspectives and professional expertise enriched, rather than constrained, the transparency and rigour of our analysis. In terms of limitations, some participants may have withheld sensitive information in a group setting. For some young people who reported a low past-year frequency of self-harm compared to lifetime, the discussion had a largely retrospective nature, which could have led to recall bias. Moreover, focus groups were conducted in 2021, during the COVID-19 pandemic, which may limit the generalisability of findings. The group spanned 16–25 years of age, which made it hard to account for possible developmental confounders, due to neurocognitive maturation and to growing autonomy, e.g., in shaping one’s goals in older compared to younger participants. It is likely that differences in age, gender, imagery ability (including possible aphantasia), varying levels of both current and past depression and self-harm behaviour influenced the group discussions. A sophisticated analysis of the interaction between individual characteristics, group dynamics, and theme generation could further enrich the interpretation of our findings. As noted above, a main limitation of our findings is that while our topic guide tried to separate general experience and to capture trait-level biases from experience directly related to self-harm, these were hard to disentangle, as was the impact of concurrent depression. It would be important to extend our findings by asking about periods of other dominant affective states (anxiety, irritability) that are often associated with self-harm, and by conducting focus groups with young people with depression but no concurrent self-harm, as well as without mental health difficulties. It is also possible that different study designs are better suited to disentangle these associations, such as ecological momentary assessment studies, in which variations over time in affect, behaviour, and motivational processes could be modelled. Other limitations included researcher bias (e.g., all female researchers) and the fact that, as this was a student project, we were not able to involve lived-experience co-researchers in all stages of the research [76].

### 4.2. Future Research and Clinical Implications

Our results indicate that interventions that enhance savouring and general future-orientation may only be relevant when self-harm presents in the context of depression and, in particular, anhedonia. Rather than learning to simply enjoy positive emotions, young people with self-harm may benefit from focusing on rewards that originate outside one’s control, such as an unexpected compliment or a lucky event, and this may be as important as learning to manage negative emotions. Our findings also suggest that improving the ability to feel in control via alternative rewarding behaviours may represent a valuable target for treatment for individuals whose self-harm is motivated by gaining control.

Future research needs to better explore the reciprocal relationship between short-term and long-term goal pursuit processes, mental imagery, and changes in self-harm behaviour and mood. Namely, self-harm could be maintained by devaluing short-term goals when euthymic and/or devaluing long-term goals when low. Overall, it is possible that developing the ability to value and be satisfied by achievable short-term goals that are in one’s control might represent an important preventative target for this population during periods of relative stability. On the other hand, during periods of self-harm, therapeutic interventions may focus on identifying adaptive short-term goals, as already included in some CBT protocols [77]. Training how to modulate motivational mental imagery to serve different goal timeframes could represent an innovative intervention in keeping with the recent growth of imagery-based therapies [35,77,78,79].

## 5. Conclusions

In summary, our study suggests that the experience of rewards and goals in young people who self-harm warrants better investigation. Future research should understand if the need for control over rewards and goals, under- and over-valuation of short vs. long-term goals, and poor use of motivational mental imagery processes is driven largely by concurrent depression and how it impacts on actual self-harm. A better comprehension of these subjective experiences and the underlying cognitive mechanisms could open novel areas of support for young people both in crisis and in routine clinical management.

## Figures and Tables

**Table 1:** Focus groups topic guide.

Enjoying and Looking Forward to Pleasant Things
What are some things you enjoy/that make you happy/that are important to you in your everyday life? How do those things make you feel? Why? Do you find things that other people tend to find rewarding such as praise, compliments, money, as rewarding or not? How do you feel about looking forward to pleasant things? Is there anything in particular, that you look forward to in the future or not? (in a few days/weeks/months/years) Do you think the experiences of enjoying and/or looking forward to pleasant things are related in any way to self-harm, or not? Have these experiences changed around periods when you were self-harming compared to periods when you weren’t, or not?
**Goals**
Can you tell us about any goals you may have for the future? (tomorrow or in ten years’ time) How is the experience of setting or achieving goals, if you do that? Why? Do you think the way you view your goals is related to self-harm or not?
**Imagining goals and pleasant things**
Can you tell us if you ever visualise things you enjoy or may look forward to? If you do, how is that experience? (e.g., frequency, feelings, consequences)

**Table 2:** Participant characteristics.

Participant Number	Gender	Age	Ethnicity	Depression Scores	Self-Harm Frequency (Lifetime)	Self-Harm Frequency (Past Year)
1	W	20	Asian/Asian British	4	324	3
2	W	18	White	34	265	50
3	W	17	White	0	100	2
4	M	19	White	8	50	10
5	M	21	White	4	missing	missing
6	W	17	White	28	50	7
7	W	19	Arab	12	48	12
8	W	18	White	28	173	17
9	NB	25	White	22	Not sure	300
10	W	16	White	26	148	64
11	W	25	White	20	478	52
12	W	16	White	26	100	50

Twelve participants numbered 1–7 (*n* = 7) were part of focus Group 1 (G1). Participants numbered 8–12 (*n* = 5) were part of focus Group 2 (G2); F = female, M = male, NB = non-binary; DASS 21—Depression = Depression and Anxiety Stress Scale 21 (Normal = 0–9, Mild depression = 10–13, Moderate depression = 14–20, Severe depression = 21–27, Extremely severe depression = 28+); self-harm frequency = number of times self-harmed in the past year.

**Table 3:** Themes and sub-themes.

Theme	Sub-Themes
Rewards need deserving	Importance of being productive Unproductive enjoyments lead to temporary pleasure and negative feelings
Keeping control	Reward anticipation only for things under their control Short-term goals—more controllable Long-term goals—both more and less controllable
High self-standards	Ambitious, unattainable, unrealistic goals Perfectionism helps stay in control High standards can generate low motivation
Trapped into long-term goals	Short-term goals not rewarding Long-term goals are preferred, but more difficult to achieve and overwhelming Long-term goals can generate low motivation
Mental imagery of long-term goals not helpful	Imagery reinforces uncertainty, fear of failure, unattainability, and frustration around long-term goals Imagery as a form of escapism Imagery is a pleasant fantasy
Self-harm alters the experience and anticipation of rewards and goals attainment	Preference shifts to short-term goals Immediate gratification Usual enjoyable activities are no longer rewarding Low motivation Self-harm becomes the goal

## Data Availability

The data presented in this study are available on request from the corresponding author due to confidentiality/data protection reasons by the ethics protocol under which the data was collected.
